# The role and therapeutic prospects of intercellular communication and RNA m^6^A modification in radiation-induced liver injury

**DOI:** 10.3389/fimmu.2026.1859858

**Published:** 2026-07-07

**Authors:** Huicong Yan, Yuhang Wang, Cunyang Guo, Yifei Du, Fangqi Tian, Ping Xu

**Affiliations:** 1 School of Public Health, Shandong Second Medical University, Weifang, Shandong, China; 2 Laboratory of Radiation-induced Diseases and Molecule-targeted Drugs, School of Food and Biomedicine, Zaozhuang University, Zaozhuang, Shandong, China; 3 School of Public Health, Binzhou Medical University, Yantai, Shandong, China

**Keywords:** exosome, hepatic fibrosis, inflammation, intercellular communication, m^6^A modification, radiation-induced liver disease

## Abstract

Radiation-induced liver injury (RILI) is a common and severe complication of radiotherapy for abdominal and thoracic malignancies. Ionizing radiation triggers widespread alterations of the N^6^-methyladenosine (m^6^A) modification landscape of hepatic RNA. Dysregulated m^6^A methylation disrupts hepatocyte homeostasis, immune cell activation and intercellular communication, and thus may contribute to the progression of RILI from acute inflammation to chronic hepatic fibrosis. Although intercellular crosstalk has been reported in RILI-related studies, its crosstalk with m^6^A modification remains poorly understood. To date, no dedicated systematic review has elaborated on the m^6^A modification-dependent mechanisms potentially underlying RILI pathogenesis. Herein, we elaborate the roles of intercellular communication (including cytokine signaling, exosome-mediated crosstalk and immune cell interactions) and m^6^A modification in RILI pathogenesis. We also summarize the combined regulatory effects for these two pathways on RILI progression, covering inflammation, hepatic fibrosis, liver regeneration and tissue repair. Additionally, we highlight the latest progress in targeted interventions against these pathways, aiming to support the development of novel therapeutic strategies. This review provides a theoretical basis for further investigating the molecular mechanisms of RILI and exploring new treatment modalities.

## 1 Introduction

Radiation-induced liver injury (RILI) represents a major dose-limiting complication of radiotherapy for abdominal and thoracic malignancies, including hepatocellular carcinoma, pancreatic cancer, and esophageal cancer. The overall incidence of RILI ranges from 6% to 66% among patients administered a cumulative radiation dose of 30–35 Gy. While stereotactic body radiation therapy (SBRT) lowers the risk of hepatic toxic injury, RILI still poses a major unmet clinical challenge (1, 2). Clinically, RILI can be divided into acute and chronic phases. Typical acute lesions develop 2 weeks to 3 months after radiotherapy and present symptoms of abdominal pain, non-icteric hepatomegaly and ascites, while isolated late-onset cases are reported up to 7 months post-radiation (1). The chronic phase, marked by progressive hepatic fibrosis and portal hypertension, emerges several months to years after radiation exposure (3–5). The onset of RILI depends on multiple factors, including radiation dose, irradiated liver volume, and pre-existing liver diseases such as cirrhosis and viral hepatitis (1, 6). Currently, effective prevention and treatment strategies for RILI remain limited. Clinical management primarily relies on rational dose constraints for normal liver tissue (e.g., restricting the mean hepatic dose to 21–32 Gy based on different fractionation regimens) and advanced radiotherapy techniques including proton and heavy ion therapy. Nevertheless, specific targeted pharmacological therapies for RILI are still unavailable (7–9). Conventional radioprotective agents including amifostine exhibit limited clinical applicability, largely attributable to systemic off-target toxicity and insufficient hepatic tropism (10). Accordingly, dissecting the molecular underpinnings of RILI and identifying druggable molecular targets are imperative for designing novel targeted therapeutic regimens.

Recent studies indicate that radiation-altered intercellular communication networks and RNA m^6^A modifications may contribute to the progression of RILI. Apart from directly inducing DNA damage in hepatocytes (11), radiation extensively remodels the hepatic intercellular communication network. The activation and dysfunction of non-parenchymal cells (e.g., hepatic stellate cells and Kupffer cells [KCs]) acts as a core regulatory hub in this pathological process. These cells remodel the hepatic microenvironment via cytokine release, exosome secretion and immune cell crosstalk, events that amplify pro-inflammatory and profibrotic signaling cascades (12, 13). Damaged hepatocytes secrete abundant paracrine factors that activate hepatic stellate cells (HSCs) and promote subsequent hepatic fibrosis mainly via DDR1-dependent signaling pathways (14). Similarly, pharmacological inhibition of CD73 mitigates liver fibrosis by suppressing HSC proliferation and myofibroblastic differentiation (15). Notably, HSC activation is an evolutionarily conserved pathological hallmark of liver fibrosis across different etiologies, including carbon tetrachloride (CCl_4_)-induced liver injury and metabolic dysfunction-associated fatty liver disease (MAFLD) (16). Collectively, these mechanistic insights into the signaling pathways regulating HSC proliferation and activation offer promising translational avenues for developing RILI-targeted therapies. Increasing evidence has demonstrated that m^6^A RNA modification plays an essential role in various pathological processes, including non-coding RNA-mediated tumor progression, establishing a link between epitranscriptomic dysregulation and multiple liver diseases (17). As a primary epitranscriptomic regulatory mechanism, m^6^A modification dynamically controls RNA splicing, stability, subcellular trafficking and translation through methyltransferase “writers” [e.g., METTL3 (18), WTAP (19)], demethylase “erasers” [e.g., ALKBH5 (20)], and binding protein “readers” [e.g., YTHDC1 (21), YTHDF2 (22), IGF2BP2 (23)]. This epitranscriptomic machinery is thought to influence cellular responses to radiation stress. For these reasons, thorough dissection of the interplay between intercellular communication and m^6^A modification is essential to unravel the intricate molecular circuitry driving RILI and discover innovative therapeutic targets.

This review focuses on the core mechanisms by which intercellular communication and m^6^A modification co-regulate RILI, and is structured into three sections.

## 2 Disorder and regulation of intercellular communication network in radiation-induced liver injury

### 2.1 Cytokine-mediated intercellular communication induced by radiation

In RILI, ionizing radiation (IR) first stimulates resident hepatic cells to release a wide range of cytokines and chemokines. These molecules serve as key mediators of intercellular communication among different liver cell populations. Distinct from physiological paracrine signals that sustain normal hepatic homeostasis, cytokines induced by IR exert strong pro-inflammatory and pro-fibrotic effects. Their abnormal upregulation breaks the homeostatic balance of intercellular crosstalk in the liver, and thus initiates RILI and drives its continuous progression.

#### 2.1.1 Inflammatory factor-mediated hepatic cell crosstalk

Inflammatory factor-driven intercellular crosstalk constitutes a core pathway that triggers early immune microenvironment dysfunction in the pathogenesis of RILI. IR directly damages hepatocytes. Damaged hepatocytes release damage-associated molecular patterns (DAMPs) including high-mobility group box 1 (HMGB1), which in turn activates adjacent liver-resident KCs (24, 25). Activated KCs robustly secrete tumor necrosis factor-α (TNF-α), a paracrine cytokine that exacerbates hepatocellular apoptosis and necrotic cell death upon binding to hepatocyte surface receptors (11). This signaling cascade establishes a positive feedback circuit linking damaged hepatocytes, activated KCs, and pro-inflammatory cytokines TNF-α and IL-1β, which exacerbates hepatic parenchymal injury and amplifies the early inflammatory cascade of RILI.

#### 2.1.2 Chemokine-cytokine-mediated immune cell crosstalk

During RILI progression, chemokines and inflammatory factors jointly mediate immune cell recruitment and amplify inflammatory cascades. Together, they build a core regulatory network that drives liver injury and remodeling of the hepatic microenvironment. Chemokines recruit circulating immune cells to the liver and mediate crosstalk between hepatic tissue and peripheral blood. In mouse models of radiation-induced liver injury, multiple chemokines including CCL2 and CXCL8 were significantly upregulated in the early phase after irradiation (26). CCL2 specifically recruits Ly6C^+^ inflammatory monocytes from peripheral blood to damaged liver regions by binding to the CCR2 receptor on the surfaces of monocytes. These infiltrating monocytes differentiate into pro-inflammatory macrophages and secrete additional TNF-α, which establishes a self-perpetuating inflammatory feedback cycle (27). Activated Kupffer cells released pro-inflammatory factors including TNF-α. These factors further elevated CXCL8 expression and facilitated neutrophil chemotaxis and transendothelial migration (28). Infiltrating neutrophils directly damaged hepatic parenchymal cells by releasing reactive oxygen species (ROS) and proteases (29).

#### 2.1.3 TGF-β-mediated hepatic stellate cell crosstalk

Transforming growth factor-β (TGF-β)-mediated intercellular crosstalk promotes RILI-related fibrosis driven by hepatic stellate cell activation. IR directly upregulated TGF-β1 expression in hepatocytes and increased the responsiveness of liver sinusoidal endothelial cells (LSECs) to TGF-β (11, 30). M2-type macrophages secreted TGF-β and PDGF to facilitate HSC activation and extracellular matrix deposition, thus accelerating fibrotic progression (31). Activated HSCs communicate with quiescent HSCs through the paracrine TGF-β1 axis. This pathway activated the intracellular Smad signaling cascade, induced the transdifferentiation of HSCs into myofibroblasts, and boosted robust extracellular matrix (ECM) synthesis, which ultimately initiated liver fibrogenesis (32, 33). Activated HSCs also release TGF-β1 via autocrine signaling. This molecule sustains the activated state of HSCs, delivers paracrine signals to adjacent LSECs, and induces LSEC capillarization. This process involves the loss of fenestrae and the formation of a new basement membrane. Such phenotypic changes raised intrahepatic vascular resistance and impaired bidirectional molecular exchange between hepatocytes and the bloodstream (34). As a key intercellular messenger linking HSCs and LSECs, TGF-β1 plays a major pro-fibrotic role, especially in the fibrotic stage of RILI.

**Figure 1:**
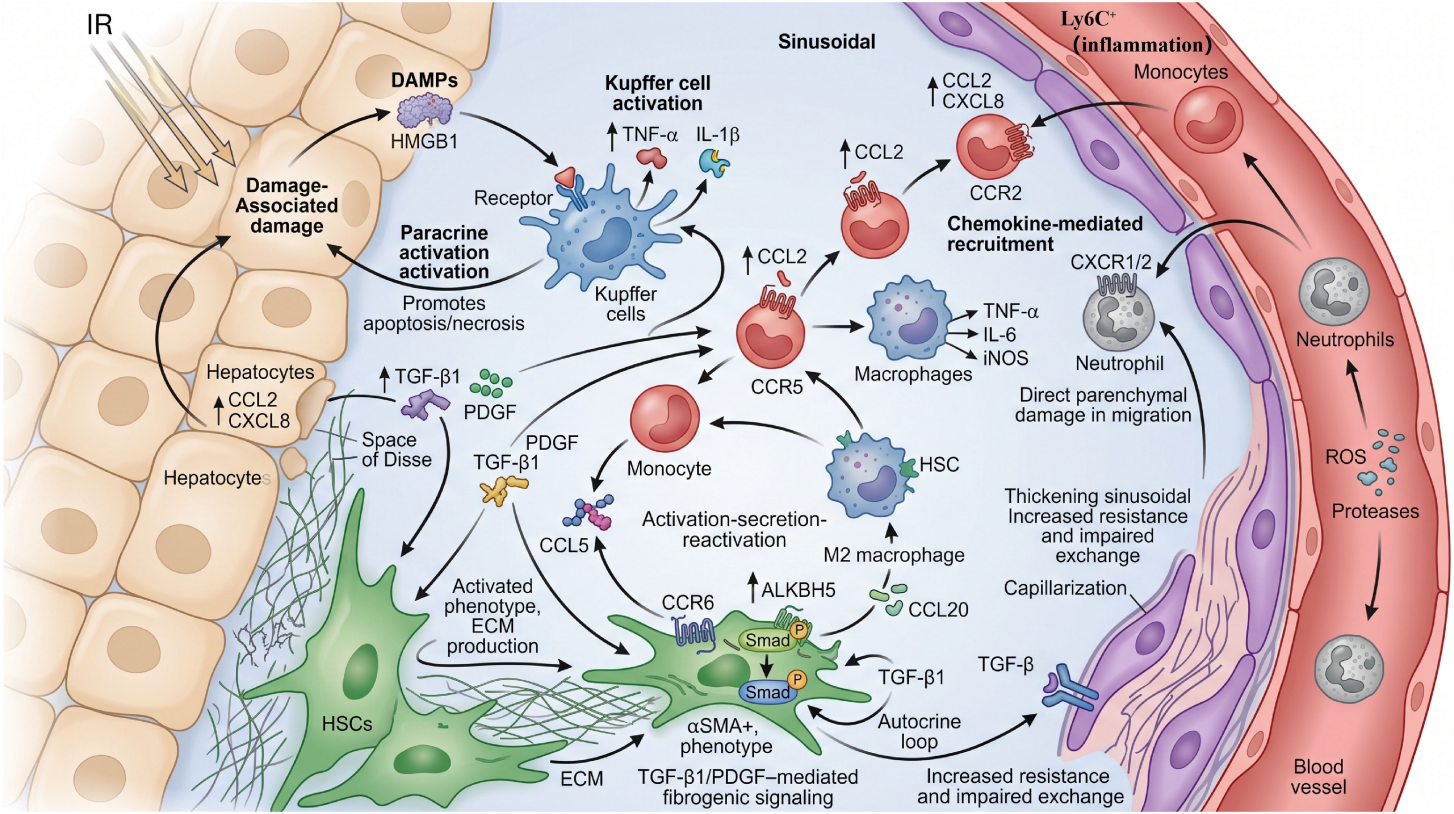
Cytokine-mediated intercellular communication network in radiation-induced liver injury.

In summary, during RILI development, ionizing radiation reshapes the intercellular crosstalk network among hepatocytes, Kupffer cells, HSCs and LSECs by inducing abnormal expression of major signaling molecules such as TGF-β, TNF-α, IL-1β, CCL2 and CXCL8. These cellular interactions go beyond simple linear signal transmission. They form a complex regulatory network with multiple positive feedback loops and mutual paracrine communication, which drives the transition of RILI from acute inflammatory damage to fibrogenesis and subsequent vascular dysfunction. Exploring these intercellular signaling pathways provides a solid theoretical basis for screening effective therapeutic targets against RILI (**Figure 1**).

### 2.2 The role of exosome-mediated intercellular communication in injury transmission and amplification

**Figure 2:**
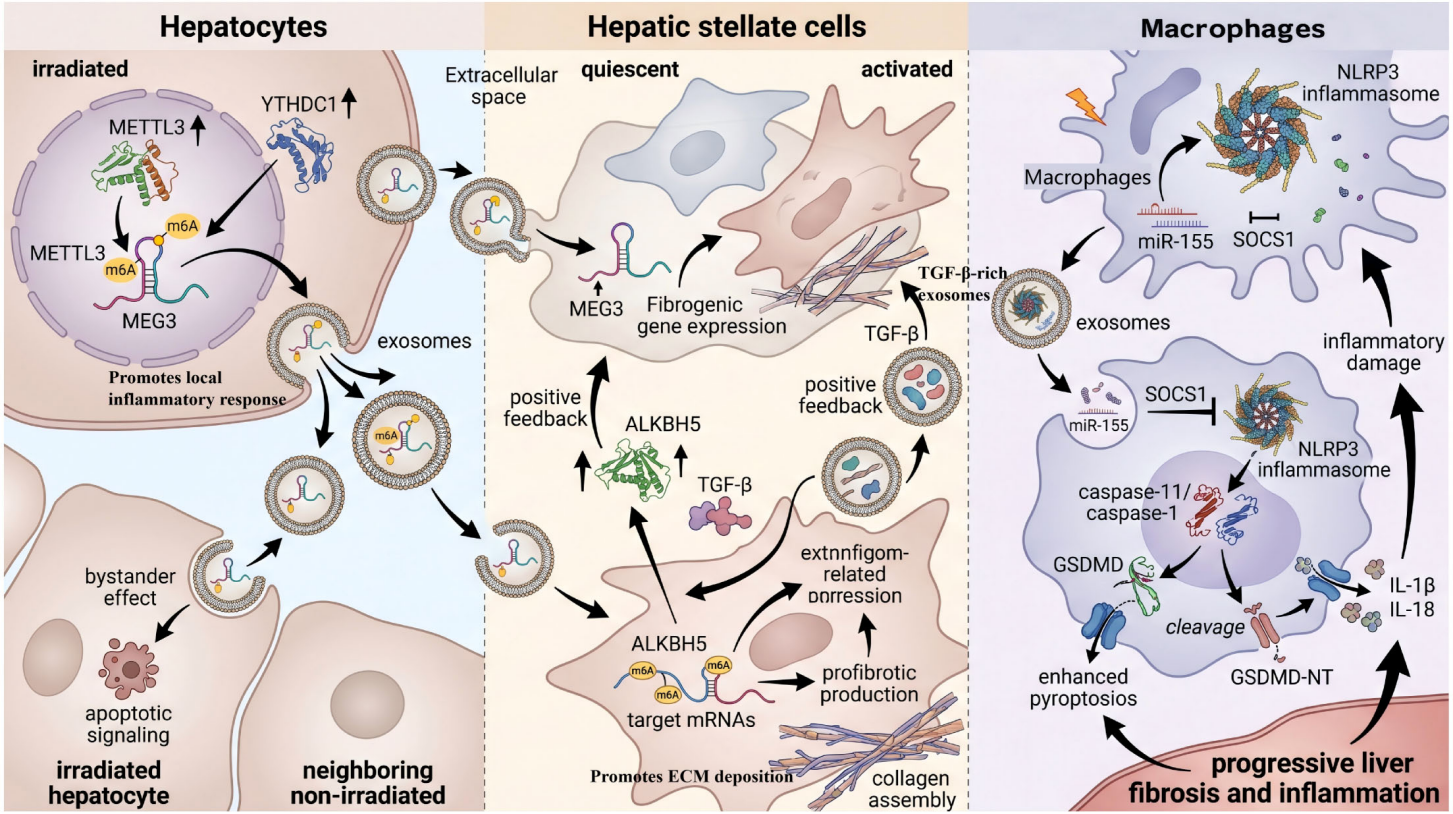
Exosome-mediated intercellular communication in radiation-induced liver injury.

In RILI, exosomes are membrane vesicles carrying bioactive molecules. They function as core regulators that propagate and amplify injury-triggered signaling cascades (21). Radiation stress substantially alters the secretion pattern and cargo composition of exosomes in different hepatic cell types. This process mediates pathological intercellular crosstalk between radiation-damaged parenchymal cells and innate immune cells (**Figure 2**).

#### 2.2.1 Hepatocyte-derived exosomes transmit injury signals

Irradiated hepatocytes release damage-related signals through exosomes. These vesicles induce radiation bystander effects in neighboring unirradiated hepatocytes and further aggravate parenchymal liver injury. Cumulative experimental evidence has shown that long non-coding RNA (lncRNA) MEG3 serves as a key molecular carrier. Its expression was tightly regulated by METTL3-YTHDC1-dependent m^6^A methylation (21). Exosomes derived from irradiated hepatocytes are rich in IncRNA MEG3. After uptake by HSCs, this RNA triggers the profibrotic activation of HSCs (21). This mechanism directly linked m^6^A writers (METTL3) and m^6^A readers (YTHDC1) to exosome-mediated crosstalk between hepatocytes and HSCs.

#### 2.2.2 Macrophage-derived exosomes amplify inflammation

Exosomes released by pyroptotic macrophages contain abundant NLRP3 inflammasome components and pro-inflammatory miR-155. Neighboring macrophages take up these exosomes, which activated the caspase-11/caspase-1-GSDMD pyroptotic cascade through modulation of the SOCS1-NLRP3 inflammasome signaling axis. This event boosted GSDMD-NT generation and the release of pro-inflammatory cytokines such as IL-1β and IL-18. It further enhanced macrophage pyroptosis and regional inflammatory activation, and ultimately drove the progression of inflammatory liver injury (35).

#### 2.2.3 HSC-derived exosomes promote fibrosis

In addition, exosomes released by activated hepatic stellate cells contain abundant fibrogenic signaling mediators (e.g., IL-11) and collagen-related proteins. These vesicles further activated quiescent hepatic stellate cells through autocrine and paracrine crosstalk loops and facilitated excessive extracellular matrix deposition (36).

Accordingly, exosomes serve as essential carriers for the long-distance transmission of injury signals throughout RILI progression. They mediate indispensable transcellular crosstalk among hepatocytes, HSCs and macrophages.

Although exosomes play crucial roles in propagating injury signals during RILI pathogenesis, several key research bottlenecks remain in current studies. Available evidence supporting exosome-mediated intercellular signal transmission is predominantly derived from *in vitro* co-culture systems, while direct *in vivo* evidence verifying intercellular exosome transfer within the hepatic microenvironment is insufficient. Future studies should establish conditional gene knockout models combined with recipient cell-specific reporter systems to validate the *in vivo* occurrence and functional significance of exosome-governed intercellular crosstalk in RILI.

### 2.3 Immune cell interaction and microenvironment remodeling

In RILI, cytokine and chemokine bursts induced by ionizing radiation ultimately trigger extensive remodeling of the hepatic immune microenvironment. This alteration mainly arises from pathological dysfunction and functional imbalance of the physiological intercellular crosstalk network across multiple hepatic immune cell subsets. Abnormal communication between immune cells not only amplifies early inflammatory responses, but also acts as a central driver for the chronic progression of RILI.

#### 2.3.1 Kupffer cell activation and inflammatory positive feedback

Kupffer cells act as the primary cellular responders to hepatic radiation injury. Following irradiation, KCs rapidly shifted from an immune-tolerant phenotype to an M1-like pro-inflammatory state, accompanied by robust secretion of TNF-α, IL-6, and IL-1β (13). These pro-inflammatory factors directly induced damage to adjacent hepatic parenchymal cells. Meanwhile, these cytokines markedly promoted the recruitment of peripheral monocytes and neutrophils to the liver by upregulating adhesion molecules (VCAM-1, ICAM-1) and chemokines (CCL2, CXCL10) on the surface of liver sinusoidal endothelial cells (LSECs) (37). Infiltrating monocytes differentiated into pro-inflammatory macrophages within the local hepatic microenvironment. This transformation further potentiated KC activation and established a positive feedback cascade that comprised KC activation, monocyte recruitment, macrophage expansion, and persistent KC reactivation.

In addition, irradiation disrupts the physiological reciprocal crosstalk between KCs and LSECs. Under homeostatic conditions, LSECs sustain KCs in a quiescent state through nitric oxide (NO) secretion. However, radiation triggered LSEC capillarization and abolished this endogenous inhibitory mechanism, thereby leading to persistent KC overactivation during RILI progression (37).

#### 2.3.2 Pathological crosstalk between macrophages and T cells

Pathological crosstalk between macrophages and T cells serves as a pivotal node that drives the transition from innate immune disturbance to adaptive immune dysregulation during RILI pathogenesis. M1 macrophages secreted IL-12 and IL-23 to facilitate the differentiation of naive CD4^+^ T cells into Th17 subsets and suppress Treg induction (38). Th17-derived IL-17A further stimulated macrophages to produce higher levels of TNF-α and IL-6, thereby forming a reciprocal pro-inflammatory circuit that amplified macrophage and Th17-mediated inflammatory responses. In contrast, M2 macrophages dominated the fibrotic stage of RILI and promoted Treg expansion by releasing the immunosuppressive cytokines IL-10 and TGF-β (39). In turn, Treg-secreted IL-35 promoted macrophage polarization toward the M2 phenotype, which established a negative immunosuppressive feedback loop to balance hepatic immune homeostasis.

Dendritic cell (DC)-T cell crosstalk also undergoes pathological dysregulation during RILI. Ionizing radiation induces intrahepatic DCs to downregulate the co-stimulatory molecule CD80 and upregulate the inhibitory molecule PD-L1. This process generates tolerogenic semi-mature DCs that failed to effectively prime naive T cells and instead promoted the differentiation of T cells into Treg or Tr1 lineages (40). Such phenotypic alterations are responsible for impaired effector T cell function and relative Treg expansion in the late stage of RILI.

TGF-β1 and IL-11 secreted by macrophages and T cells acted as core signaling mediators that drove HSC activation (36). Activated HSCs transdifferentiated into myofibroblasts, synthesized abundant type I collagen and fibronectin, and initiated ECM deposition.

#### 2.3.3 Dysregulated immune intercellular communication remodels the hepatic microenvironment

Pathologically disordered intercellular communication among multiple immune cell populations synergistically drives comprehensive remodeling of the hepatic immune microenvironment, a process that determines the overall progression trajectory of RILI.

Pro-inflammatory remodeling of the microenvironment dominates the early phase of RILI. Continuous tissue infiltration by M1 macrophages, Th17 cells and neutrophils, alongside elevated release of TNF-α, IL-6 and CXCL8, sustained sterile hepatic inflammation (41). This inflammatory milieu promoted the generation of ROS and nitric oxide (NO), provoked mitochondrial dysfunction and hepatocyte apoptosis, and formed a positive feedback cycle of “injury-tissue damage-worsened inflammation”.

The pro-fibrotic microenvironment serves as a characteristic pathological signature of chronic RILI. Driven by fibrogenic TGF-β1 and PDGF, HSCs stayed chronically activated and secreted abundant extracellular matrix (ECM). Meanwhile, M2 macrophages suppressed ECM breakdown by secreting tissue inhibitors of metalloproteinases (TIMPs), rendering fibrotic accumulation irreversible (42). Such a pro-fibrotic microenvironment further compressed hepatic sinusoids and exacerbated local hypoxia. Hypoxia in turn reactivated HSCs and macrophages, establishing a self-perpetuating profibrotic feedback loop.

The immunosuppressive hepatic microenvironment underlies widespread adaptive immune dysfunction. Chronic antigen stimulation and sustained pro-inflammatory stress upregulate immune checkpoint molecules (e.g., PD-1 and Tim-3) on CD8^+^ cytotoxic T cells. This alteration induced progressive T cell exhaustion and reduced the secretion of cytotoxic effector molecules (43, 44). In addition, radiation markedly elevates PD-L1 expression on hepatocytes and liver sinusoidal endothelial cells. Surface PD-L1 robustly suppressed the effector function of infiltrating T cells through the PD-1/PD-L1 signaling axis (45).

Exhausted CD8^+^ cytotoxic T cells lose the capacity to eliminate radiation-damaged hepatocytes, thereby increasing the susceptibility to liver cirrhosis and subsequent hepatocellular carcinoma. Notably, radiation-induced senescent hepatocytes exhibit a senescence-associated secretory phenotype (SASP), which features substantial secretion of pro-inflammatory IL-6. This cytokine upregulates surface CD73 expression in neighboring hepatic macrophages via the JAK/STAT3 signaling axis, leading to interstitial adenosine accumulation. Such metabolic alterations established a novel metabolic immune checkpoint that suppressed the anti-damage and anti-tumor effector activities of CD8^+^ T cells (46, 47).

**Figure 3:**
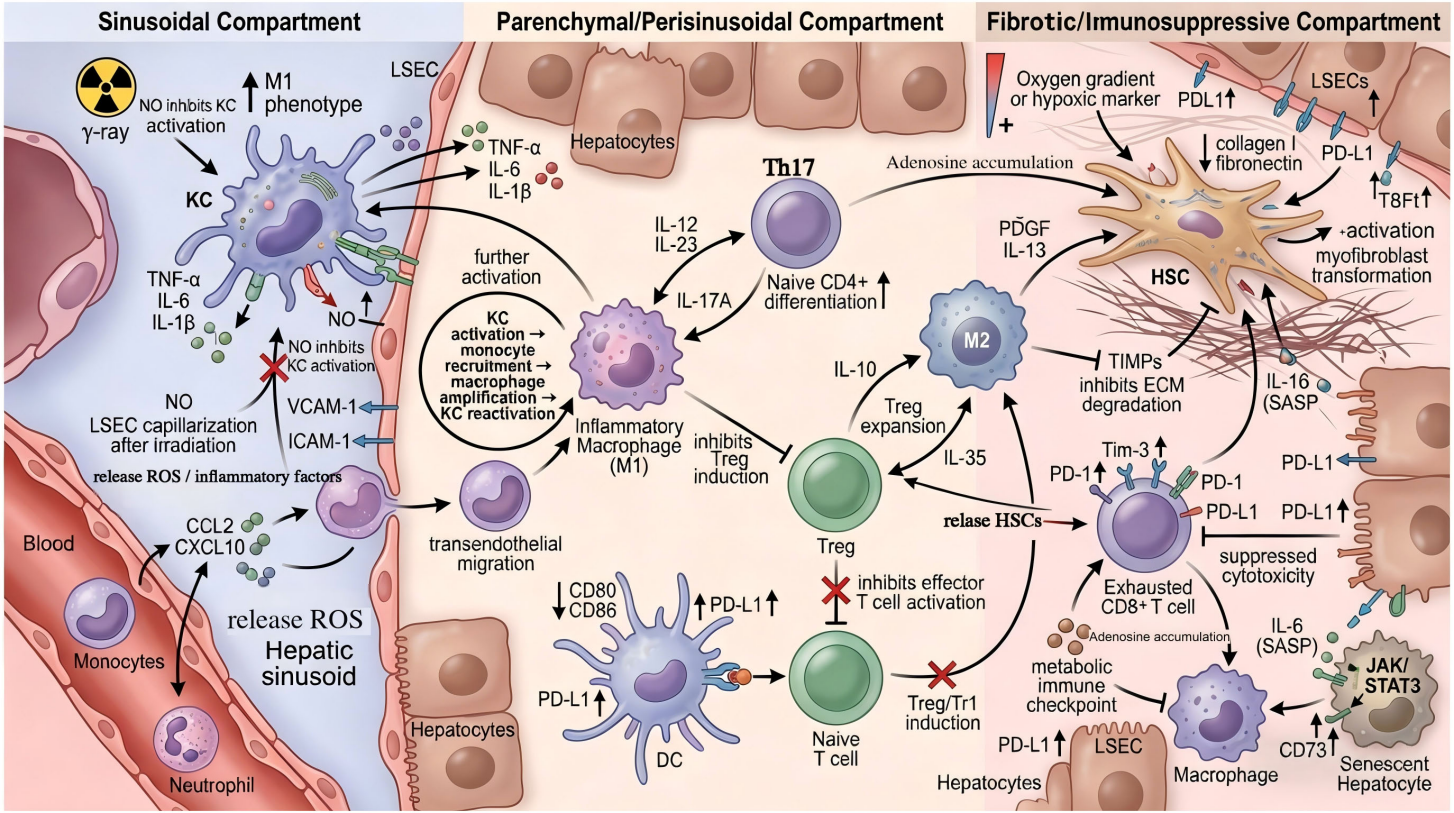
Immune cell crosstalk and microenvironment remodeling in radiation-induced liver injury.

In conclusion, pathological disruption of immune cell crosstalk throughout RILI pathogenesis initiates a progressive signaling cascade that bridges innate immune activation and adaptive immune dysfunction, thereby driving the transition from acute hepatic inflammation to persistent fibrogenesis. Multiple pathological events, including M1 polarization of KCs, macrophage-Th17 pro-inflammatory loops, macrophage-HSC positive feedback circuits, and T cell exhaustion, jointly remodel the hepatic immune microenvironment. These aberrant interactions construct an integrated pathological axis covering persistent inflammation, progressive fibrogenesis, and local immunosuppression. This disordered microenvironment not only facilitates the progression of acute radiation hepatic injury toward chronic liver fibrosis but also provides solid theoretical support for the development of novel therapeutic strategies targeting key immune crosstalk nodes in RILI treatment (**Figure 3**).

## 3 Regulatory functions and mechanisms of RNA m^6^A modification in radiation-induced liver injury

**Figure 4:**
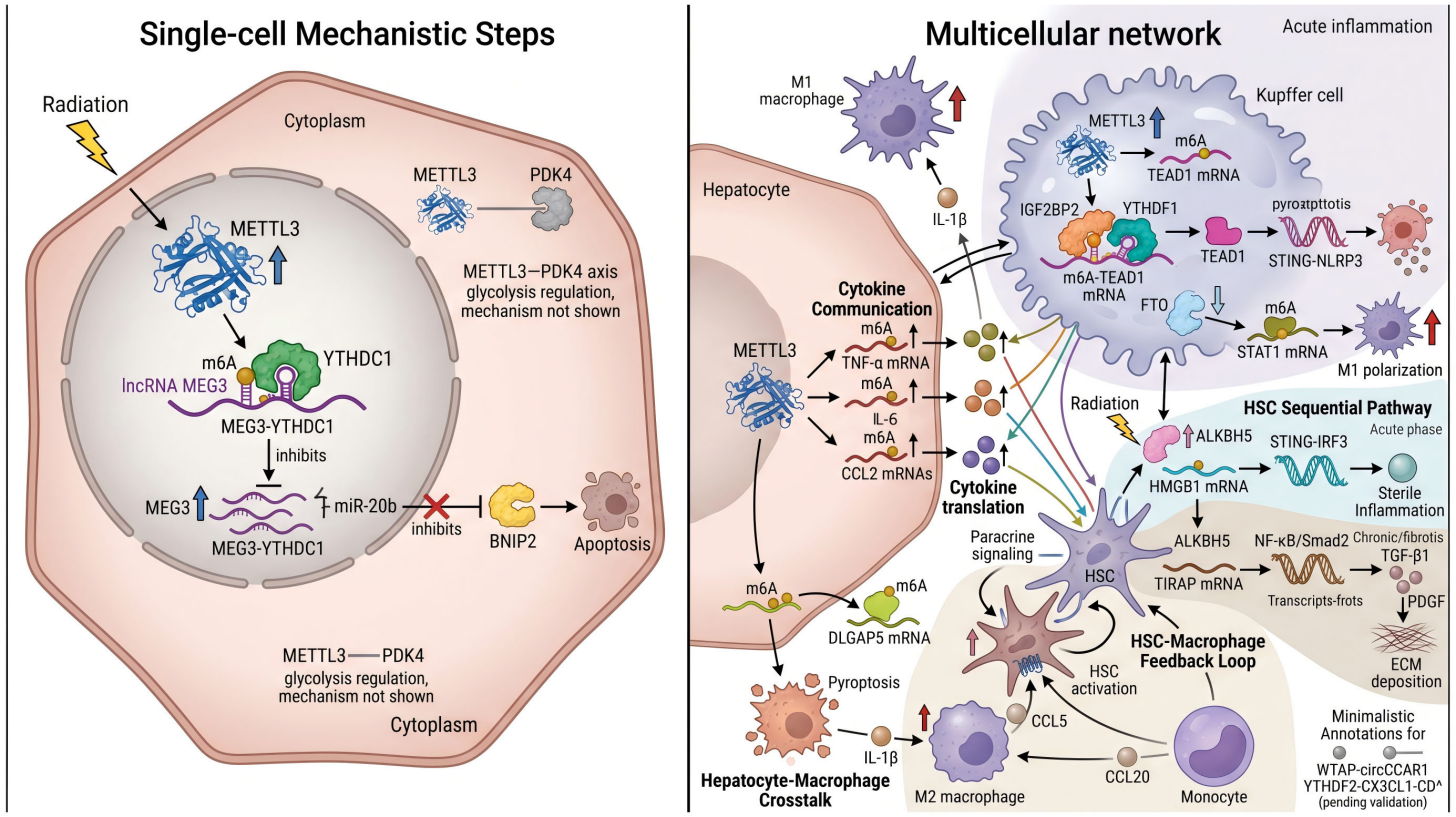
Regulatory mechanism of RNA m^6^A modification in radiation-induced liver injury.

Aberrant expression of cytokines, exosomal cargos, and immune polarization markers during RILI has been linked to upstream m^6^A modification machinery. This section systematically elaborates how ionizing radiation may remodel the expression profiles and enzymatic activities of m^6^A writers, erasers, and readers in multiple hepatic cell populations. These molecular alterations could potentially reconfigure the core intercellular crosstalk nodes summarized in Chapter 2 by regulating the stability and turnover of downstream target mRNAs. Three typical signaling axes are emphasized in this chapter, including TNF-α-mediated positive feedback loops, exosome-dependent MEG3 transmission, and the ALKBH5-governed signaling circuit between macrophages and hepatic stellate cells (**Figure 4**). By clarifying the hierarchical association among m^6^A modification, signaling molecule expression, and rewired cellular communication, this chapter aims to establish a theoretical framework for the epitranscriptome-cell interaction mechanism underlying RILI pathogenesis.

The m^6^A modification pattern was synergistically orchestrated by methyltransferase complexes (writers, including METTL3, METTL14, and WTAP), demethylases (erasers, including FTO and ALKBH5), and m^6^A reader proteins (readers, such as YTH domain-containing proteins and the IGF2BP family) (48). This epigenetic mark modulates multiple mRNA metabolic processes, including splicing, nuclear export, transcript stability, and translational efficiency, thereby participating in a wide range of physiological and pathological events such as cell apoptosis, proliferation, inflammatory activation, and phenotypic differentiation. Ionizing radiation triggers extensive remodeling of the hepatic m^6^A epitranscriptomic landscape. As a central upstream regulatory hub, dysregulated m^6^A signaling drives the progression of RILI from acute inflammatory damage to chronic fibrosis by precisely modulating hepatocyte homeostasis, immune cell activation, and parenchymal intercellular crosstalk. **Table 1** comprehensively summarizes the current evidence linking m^6^A modification enzymes, downstream target molecules, and aberrant intercellular signaling during RILI progression.

**Table 1:** Correlation among m^6^A methyltransferases, target genes, and communication effects.

m^6^A enzyme	Cell type	Target gene/molecule	m^6^A effect	Communication outcome	Evidence level
METTL3	Kupffer cells	TNF-α mRNA	Stability ↑	Hepatocyte-KC positive feedback amplification	Directly confirmed in RILI (48)
METTL3	Hepatocytes	MEG3 lncRNA	Stability ↑ (recognized by YTHDC1)	Exosome-mediated hepatocyte→HSC activation	Directly confirmed in RILI (21, 49)
METTL3	Hepatocytes	PDK4 mRNA	Translation efficiency ↑	Metabolic reprogramming (indirectly related to communication)	Other models (50)
ALKBH5	HSCs	TIRAP mRNA	Demethylation→ Stability ↑	TGF-β1 paracrine→Macrophage-HSC positive feedback	Directly confirmed in RILI (51)
ALKBH5	HSCs	HMGB1 mRNA	Demethylation→ Stability ↑	STING pathway activation→Inflammation amplification	Directly confirmed in RILI (52)
YTHDF1	Kupffer cells	NLRP3 mRNA	Translation efficiency ↑	Pyroptotic exosomes→Inflammation amplification	Indirect in RILI (53)
YTHDC1	Hepatocytes	MEG3	Recognize m^6^A→Stabilization	Exosome loading→HSC activation	Directly confirmed in RILI (21)
IGF2BP2	Kupffer cells	TEAD1 mRNA	Recognize m^6^A→Stabilization	STING-NLRP3 pyroptosis	Indirect in RILI (54)

→: signal transduction and cascade activation. ↑: Up-regulated expression or elevated molecular levels.

### 3.1 Direct regulation of hepatocyte injury and repair by RNA m^6^A modification

Direct epitranscriptomic regulation of hepatocyte injury and repair mediated by m^6^A modification is suggested to constitute the initial pathological event during RILI pathogenesis. These cell-autonomous processes occur primarily within hepatocytes, independent of intercellular crosstalk, and may prime the hepatic microenvironment for subsequent inflammatory cascades and fibrotic progression.

The m^6^A machinery directly modulates hepatocyte damage and regenerative capacity, thereby forming the upstream pathological trigger of RILI. In terms of hepatocyte apoptosis, METTL3 deposits m^6^A marks on long non-coding RNA MEG3. The modified transcript is subsequently recognized by the reader protein YTHDC1, which stabilized MEG3 and elevated its endogenous expression. This regulatory cascade abolished the inhibitory effect of miR-20b on the pro-apoptotic gene BNIP2, ultimately aggravating radiation-induced hepatocyte apoptosis (21, 51). Beyond apoptotic signaling, m^6^A modification also participates in the metabolic regulation of irradiated hepatocytes. Specifically, the METTL3-PDK4 axis rewired glycolytic metabolism and reshaped cellular metabolic homeostasis following radiation exposure (50). Nevertheless, the functional implications of hepatocyte metabolic reprogramming for parenchymal intercellular crosstalk during RILI remain poorly elucidated and require further validation. In addition, whether m^6^A modification modulates lipid metabolism, amino acid metabolism, and mitochondrial function in radiation-injured hepatocytes still lacks systematic exploration under RILI pathological conditions.

### 3.2 RNA m^6^A modification regulates intercellular communication in RILI

Beyond the direct modulation of hepatocyte injury and repair, m^6^A modification acts as a critical upstream regulatory module that governs intercellular crosstalk throughout RILI pathogenesis. This epitranscriptomic regulatory cascade covers multiple pathological communication modes, including cytokine signaling transmission, exosomal cargo delivery, immune cell interactive crosstalk, and HSC profibrotic activation.

#### 3.2.1 Cell type- and disease stage-specificity of m^6^A modification

Accumulating evidence has suggested that m^6^A regulatory factors function in a strictly cell-type-specific and stage-dependent manner during radiation-induced liver injury. Such dynamic context-dependent characteristics are particularly evident for METTL3 and ALKBH5, which exert antagonistic functions across the acute inflammatory and chronic fibrotic stages of RILI.

The biological functions of METTL3 exhibit strict cell-type specificity and disease stage dependence. During the acute phase of RILI, METTL3 expression is downregulated in irradiated hepatocytes. This METTL3 depletion protects hepatocytes from radiation-induced apoptosis by reshaping the m^6^A methylation landscape of pro-apoptotic target transcripts (49). Conversely, KCs upregulate METTL3 expression during the same acute stage (54). METTL3-mediated m^6^A modification enhanced TEAD1 mRNA stability, thereby activating the STING-NLRP3 inflammasome cascade, facilitating macrophage pyroptosis, and promoting the release of pro-inflammatory cytokines (55). The reader proteins IGF2BP2 and YTHDF1 bound and stabilized methylated TEAD1 mRNA to augment NLRP3 translation, further amplifying intrahepatic pro-inflammatory signaling (53).

In chronic liver injury models such as primary sclerosing cholangitis, hepatocyte-specific METTL3 knockout exacerbated biliary damage and progressive hepatic fibrosis (56). These findings highlight the stage-dependent dual roles of hepatocytic METTL3. Specifically, METTL3 downregulation conferred hepatoprotective effects during acute RILI, whereas sustained METTL3 deficiency aggravated fibrotic progression in the chronic stage. Collectively, METTL3 may exert opposing biological functions within the same cell type at different disease stages, driving the phenotypic transition from acute inflammation to chronic fibrosis.

During the acute inflammatory stage of RILI, ALKBH5 was upregulated in HSCs. ALKBH5 stabilized HMGB1 transcripts by erasing m^6^A methylation within the 3′ untranslated region (3′UTR) of HMGB1 mRNA, thereby activating the STING-IRF3 signaling cascade and triggering robust sterile hepatic inflammation (51). In the chronic fibrotic stage, accompanied by decreased HMGB1 expression and sustained HSC activation, ALKBH5 preferentially stabilizes TIRAP mRNA. This epitranscriptomic modification synergistically potentiated NF-κB/Smad2 signaling, facilitated excessive extracellular matrix deposition, and ultimately accelerated hepatic fibrotic progression (52). Collectively, ALKBH5 may have dual pro-inflammatory and pro-fibrotic functions in a stage-dependent manner, driving acute inflammatory responses and chronic fibrogenesis during RILI progression.

Taken together, these findings indicate that m^6^A regulators, including METTL3, ALKBH5, IGF2BP2, and YTHDF1, exhibit highly dynamic and context-dependent functions strictly governed by cell type and disease stage. Accordingly, global pharmacological inhibition of a single m^6^A enzyme rarely yields optimal therapeutic outcomes and may even induce adverse hepatic effects by disturbing physiological signaling in distinct hepatic cell populations at inappropriate disease stages. Therefore, future therapeutic strategies for RILI should adopt cell-selective and stage-specific intervention paradigms.

Specifically, targeted inhibition of METTL3 in KCs alleviated acute intrahepatic inflammatory infiltration, whereas hepatocyte-specific METTL3 suppression conferred potent anti-apoptotic protection on hepatic parenchymal cells. Moreover, ALKBH5 blockade in acute RILI attenuated excessive hepatic inflammatory cascades, whereas specific ALKBH5 downregulation at the chronic fibrotic stage effectively arrested progressive hepatic fibrogenesis.

#### 3.2.2 m^6^A-mediated intercellular communication mechanisms

RNA m^6^A methylation has been proposed as a crucial upstream regulatory module that may orchestrate RILI pathogenesis by influencing the complex hepatic intercellular communication network. In terms of cytokine-dependent crosstalk, m^6^A modification profoundly modulates the transcription and extracellular secretion of pro-inflammatory and profibrogenic mediators in the liver. In HSCs, ALKBH5 erased m^6^A methylation from TIRAP transcripts to activate NF-κB signaling cascades, thereby potentially promoting the production of fibrogenic factors including TGF-β1 and PDGF. These cytokines may act on neighboring HSCs and macrophages through paracrine signaling, ultimately contributing to a self-amplifying positive feedback loop during RILI progression (51).

Moreover, m^6^A modification controls hepatic injury signal transmission by regulating exosomal transcript stability and cargo packaging. In hepatocellular carcinoma, WTAP-mediated m^6^A modification enhanced circCCAR1 stability and facilitated its exosomal encapsulation, thereby inducing T cell dysfunction (57). However, this regulatory paradigm remains unvalidated in the RILI context. With respect to macrophage polarization, radiation-induced METTL3 downregulation led to STAT1 mRNA hypomethylation, which drove M1 pro-inflammatory polarization of KCs (13, 58). Conversely, ALKBH5 upregulation in M2 macrophages sustained persistent HSC activation by stabilizing the transcripts of fibrogenic mediators (51).

M2 macrophage-derived CCL5 further recruited peripheral monocytes and promoted their phenotypic transition toward the M2 phenotype. These M2-polarized macrophages subsequently secreted CCL20, which bound to the CCR6 receptor on HSCs to further elevate ALKBH5 expression. This cascade formed a self-sustaining “activation-secretion-reactivation” positive feedback circuit that perpetuated hepatic fibrogenesis (51). In liver cancer models, YTHDF2 reinforced CD8^+^ T cell infiltration by stabilizing CX3CL1 mRNA (59). Although m^6^A-dependent modulation of immune checkpoint molecules (e.g., PD-1 and CTLA-4) has been well documented in tumor immunity (60), direct evidence supporting such regulatory mechanisms in RILI remains limited.

Furthermore, hepatocyte pyroptosis during acute RILI drove macrophage metabolic reprogramming and subsequent M1 polarization via paracrine release of pro-inflammatory cytokines such as IL-1β. Mechanistically, METTL3-dependent m^6^A methylation of DLGAP5 served as a core upstream trigger of hepatocyte pyroptosis (61), indicating that m^6^A epitranscriptomic modification exacerbated RILI-related inflammatory injury through the hepatocyte-macrophage crosstalk axis.

In summary, relying on the coordinated function of m^6^A writers, erasers, and readers, m^6^A RNA methylation comprehensively modulates acute injury and chronic fibrotic progression of RILI via two core mechanisms: the direct regulation of hepatocyte injury and repair, and the epitranscriptomic orchestration of multi-cell-type intercellular crosstalk.

## 4 Intervention strategies targeting cell communication and m^6^A modification and their clinical feasibility analysis

### 4.1 Pharmaceutical intervention

Targeting pivotal nodes within the hepatic intercellular crosstalk network represents a promising therapeutic strategy for ameliorating RILI. Current progress in RILI intervention has primarily covered three core therapeutic avenues including blockade of major chemokine signaling axes, suppression of fibrosis-associated cascades, and modulation of oxidative stress and apoptotic pathways.

Disrupting chemokine-receptor signaling axes serves as a straightforward approach to break pro-fibrotic positive feedback loops during RILI progression. Mechanistically, ALKBH5-dependent demethylation of TIRAP initiated the CCL5-CCR6-CCL20 pro-fibrotic cascade, which acted as a central driver of RILI-associated hepatic fibrosis (51). Preclinical studies demonstrated that combined CCR6 inhibition and ALKBH5 knockdown markedly alleviated radiation-induced liver fibrosis and enhanced the radiosensitivity of hepatocellular carcinoma (51). In addition, CCR5 blockade or neutralization of its ligand CCL5 exerted robust anti-fibrotic efficacy in multiple liver fibrosis models (62). Collectively, these signaling axes provide reliable targets for precise intervention against fibrotic-stage RILI.

As efficient bioactive delivery vehicles, exosomes exhibit considerable translational potential for RILI treatment. In preclinical liver fibrosis models, mesenchymal stem cell-derived exosomes carrying miR-27b-3p effectively suppressed HSC activation and alleviated fibrotic lesions (63). Moreover, CD44-targeted exosomal delivery of forsythiaside A mitigates NLRP3 inflammasome-mediated hepatocellular pyroptosis and relieves hepatic inflammatory injury (64). Nevertheless, the therapeutic efficacy of these exosome-based strategies in RILI remains to be further validated. Notably, emerging liver-targeted modification and delivery technologies have greatly facilitated the clinical translation of engineered exosome therapeutics.

In addition, smart hydrogel-based sustained-release systems loaded with effector memory T cells and PD-1 inhibitors enable precise lymph node targeting, reshape the local immune microenvironment, and enhance systemic anti-tumor immunity in preclinical tumor models (65). Such multifunctional delivery systems provide novel insights for the development of combinatorial intervention strategies applicable to RILI treatment.

### 4.2 Exploration of precise therapy targeting m^6^A modifying enzymes

Therapeutic targeting of core m^6^A writer, eraser, and reader proteins provides novel strategies for precise intervention against RILI.

Interventions targeting m^6^A methyltransferases (writers) have been the most extensively investigated therapeutic avenue. In RILI, METTL3 mediates YTHDC1-dependent m^6^A modification of MEG3, thereby modulating the inhibitory effect of miR-20b on the pro-apoptotic gene BNIP2. Therapeutic strategies targeting this METTL3-associated regulatory axis, including METTL3 small interfering RNA transfection and YTHDC1 inhibitor administration, protected hepatocytes and suppressed radiation-induced hepatocellular apoptosis in RILI models (21). These findings demonstrate that targeted modulation of the METTL3-YTHDC1-MEG3 axis serves as a promising therapeutic approach for RILI. In addition, the selective METTL3 inhibitor STM2457 alleviates mitochondrial damage and ameliorates metabolic dysfunction-associated fatty liver disease in preclinical studies (49).

Targeting the demethylase ALKBH5 constitutes another feasible therapeutic strategy for RILI. ALKBH5 activates the STING signaling pathway via HMGB1 mRNA demethylation, which facilitates innate immune activation and exacerbates hepatocyte apoptosis during RILI pathogenesis. ALKBH5 knockdown abrogated HMGB1-dependent STING activation and mitigated intrahepatic inflammatory responses (52). Blockade of the ALKBH5-CCR6 axis, achieved via either CCR6 small-molecule antagonism or ALKBH5 siRNA interference, markedly ameliorated radiation-triggered hepatic fibrosis and improved the radiosensitivity of hepatocellular carcinoma cells (51). These findings support ALKBH5 as a core therapeutic target for RILI-related fibrosis, whereas specific small-molecule inhibitors targeting ALKBH5 remain under preclinical development. The FTO inhibitor CS2 inhibits the proliferation and migration of hepatocellular carcinoma cells (66), and FTO-targeted proteolysis-targeting chimera (PROTAC) technology has also been validated in metabolic dysfunction-associated fatty liver disease models (67). Nevertheless, the efficacy, safety profile and therapeutic window of these candidate agents in the context of RILI remain poorly characterized, which necessitates systematic preclinical validation.

### 4.3 Multi-target combination therapy and future research directions

**Figure 5:**
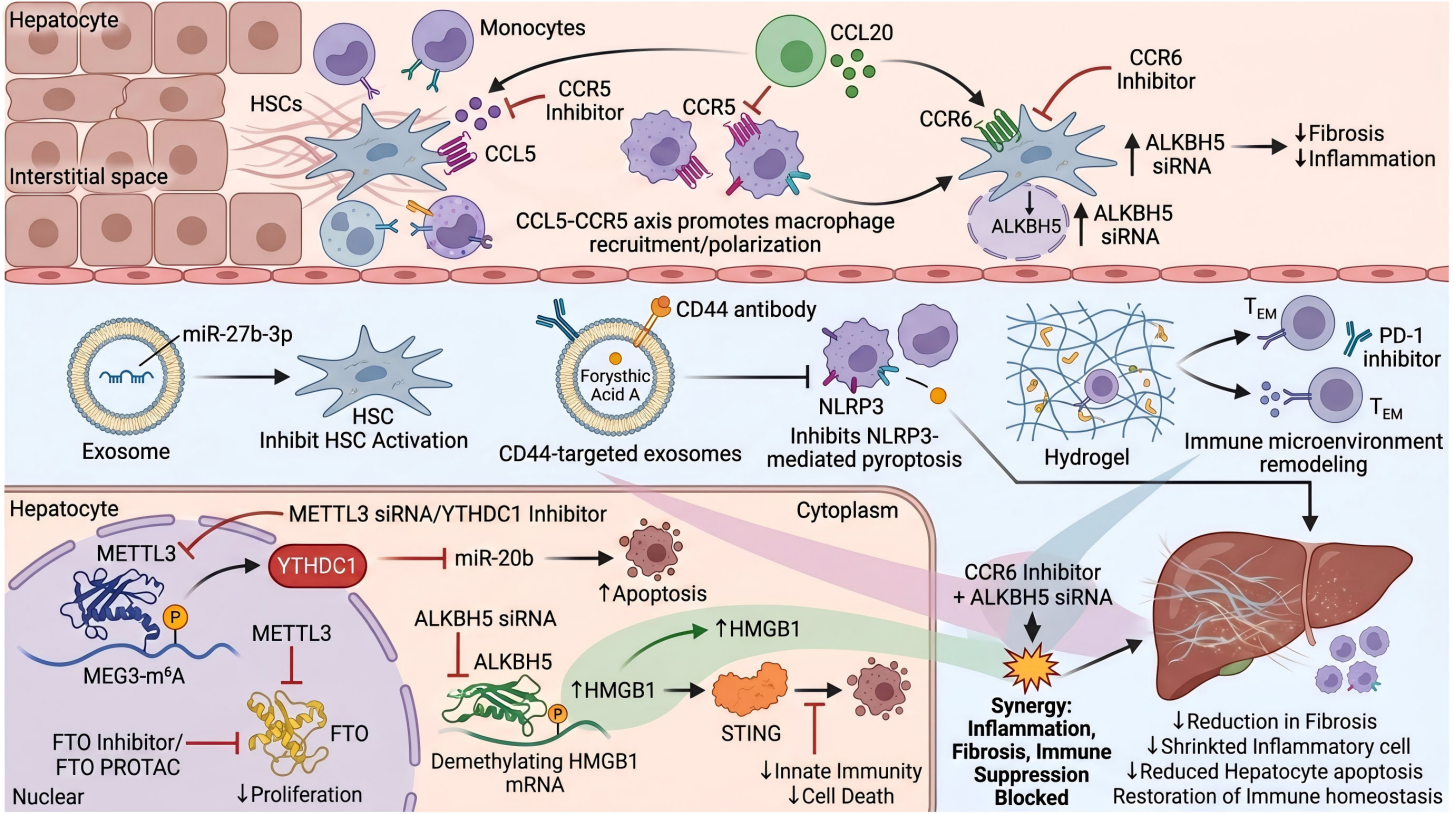
Intervention strategies and combination therapy targeting cellular communication and m^6^A modification for radiation-induced liver injury.

Interventions against a single molecular target cannot fully interrupt the intricate pathological network driving RILI progression, whereas multi-target combinatorial therapeutics are expected to improve therapeutic efficacy and overcome drug resistance through synergistic regulatory effects. Such combinatorial regimens include m^6^A-targeted methylation modulators, antibody-based therapeutics, and other strategies engineered to remodel intercellular communication. In immunotherapy studies of hepatocellular carcinoma, combined treatment with the METTL3 inhibitor STM2457 and anti-PD-1 antibodies substantially boosted the infiltration and effector functions of CD8^+^ T cells, which in turn reversed the immunosuppressive hepatic microenvironment (68). Concurrent blockade of chemokine signaling axes including the CCR2 and TGF-β1 cascades alleviates inflammatory cell infiltration and arrests fibrogenesis in metabolic dysfunction-associated steatohepatitis (MASH) models (69). In preclinical alcoholic liver disease models, lipid nanoparticle-encapsulated anti-miR-96 combined with the Hedgehog pathway inhibitor MDB5 exerts synergistic inhibitory effects on HSC activation and collagen deposition (70). Collectively, these preclinical findings suggest that combinatorial targeting strategies may enable simultaneous modulation of multiple core pathological nodes throughout the stages of inflammatory initiation, fibrotic progression and local immunosuppression in RILI. Sections 4.1, 4.2 and 4.3 are illustrated in (**Figure 5**).

### 4.4 Clinical feasibility analysis

Comprehensive assessment of multiple core factors is essential to validate the clinical translational potential of therapeutic regimens targeting m^6^A modification and intercellular crosstalk in RILI. These factors include drug safety, administration routes, optimal therapeutic windows, and combined medication strategies.

From the perspective of clinical translation and medication safety, existing m^6^A-targeted agents pose prominent potential risks to clinical application. Most m^6^A regulatory compounds such as STM2457 (71) and ALKBH5 siRNA (72) lack complete toxicological data regarding normal hepatic tissues. Since m^6^A modification universally modulates diverse RNA metabolic processes, systemic inhibition of ALKBH5 may disrupt hepatocyte regeneration, lipid metabolism and immune homeostasis, and further induce unexpected off-target toxic effects. Accumulating experimental data verify that ALKBH5 exerts protective functions against hepatic ischemia-reperfusion injury in various preclinical models, which raises vital concerns that ALKBH5 inhibition could aggravate acute inflammatory lesions during RILI progression (48). Epitranscriptomic modulators must undergo strict safety and toxicology testing prior to clinical translation, with special attention paid to normal hepatic, renal and nervous tissues (73).

Agents designed to disrupt pathological intercellular crosstalk, such as CCR5 (74) and CCR6 antagonists (75), exhibit acceptable safety performance in the treatment of other chronic liver disorders. Even so, their clinical use for RILI demands additional evaluation to rule out possible impairment of anti-tumor immune surveillance against residual malignant cells. For example, although CCR5 blockade effectively relieves hepatic fibrosis, it may simultaneously hinder the recruitment of tumor-suppressive CD8^+^ T lymphocytes (76).

Oral administration exhibits prominent clinical accessibility and serves as the most prevalent administration route for routine clinical drugs. However, most m^6^A modulators, including the METTL3 inhibitor STM2457 (72) and the FTO inhibitor CS2 (77), are highly polar heterocyclic compounds. These inherent physicochemical characteristics render such agents prone to intensive hepatic first-pass metabolism, which substantially impairs their systemic *in vivo* bioavailability. In comparison, engineered exosomes represent sophisticated and high-efficiency drug delivery platforms. Equipped with surface modification strategies such as CD44 targeting, exosomes achieve precise liver- and cell-specific drug delivery, thereby effectively circumventing the inherent limitations of conventional administration modalities (64).

Beyond direct therapeutic cargo transportation, exosomes function as ideal delivery vehicles for targeted hepatic intervention. Accumulating preclinical evidence has demonstrated that exosome-based targeted delivery systems combined with vancomycin exerted enhanced anti-inflammatory efficacy and reduced systemic toxicity in liver abscess models. These findings provide solid conceptual support for the clinical application of engineered exosome-mediated hepatic delivery of anti-inflammatory agents (78). Furthermore, gradient crosslinked adhesive patches based on polydimethylsiloxane (PDMS) facilitate the repair of damaged hepatic tissue and show promising potential for liver injury treatment (79).

The temporal dynamics of m^6^A modification and intercellular crosstalk jointly dictate the optimal intervention window for RILI treatment. Hepatic m^6^A epitranscriptomic profiles underwent dramatic changes within 24–48 hours following radiation exposure (49). Specifically, radiation exposure markedly downregulates the methyltransferase METTL3 at two days post irradiation, reduces global m^6^A levels, and subsequently initiates a cascade of hepatocellular apoptotic events (49). In the early stage of RILI, the m^6^A reader YTHDC1 is recruited to the long non-coding RNA MEG3. This molecular event modulates mitochondrial autophagy and cellular apoptosis via the miR-20b/BNIP2 signaling axis, which provides a critical temporal basis for early prophylactic intervention against RILI (21).

Accordingly, prophylactic treatment with YTHDC1 inhibitors should preferably be delivered within 24 hours before radiation exposure, and early therapeutic intervention within 24–48 hours after irradiation should be recommended to maximize treatment efficacy. As the core initiators of RILI pathogenesis, hepatic macrophages rapidly secrete abundant pro-inflammatory cytokines, including TNF-α, IL-1β and CCL2, immediately after radiation insult. This inflammatory cascade further promotes HSC activation and drives subsequent hepatic fibrotic progression (28). Macrophage-HSC crosstalk induces metabolic reprogramming and autophagy activation primarily at 1–2 weeks after irradiation (28). Therefore, anti-inflammatory strategies targeting TNF-α, IL-1Ra, or CCR2 antagonists are suitable for administration within zero to three days post irradiation, whereas interventions against metabolic reprogramming, such as the dual CCR2/CCR5 antagonist cenicriviroc, require continuous administration from three to fourteen days after radiation exposure.

Bidirectional regulatory crosstalk occurs between molecules governing intercellular communication and m^6^A epitranscriptomic regulators. Preliminary data collected from radiation-triggered hepatic fibrosis models indicated that combined intervention via ALKBH5 silencing and CCR6 antagonists, where CCR6 functions as the receptor for CCL20, yielded synergistic anti-fibrotic activity and elevated the radiosensitivity of hepatocellular carcinoma cells (47). Nevertheless, these findings are derived exclusively from preclinical models, and whether such combinatorial strategies can achieve effective and safe outcomes in human RILI remains to be determined.

Few research works so far have explored the synergistic application of m^6^A-targeted compounds and classic radioprotective reagents such as amifostine to treat RILI, despite the inherent potential for coordinated protective effects brought by this combinatorial pattern. Amifostine eliminates reactive oxygen species (ROS) inside hepatocyte mitochondria and recovers normal autophagic flux to mitigate RILI-related tissue damage. This reagent fails to generate obvious radioprotective impacts on hepatocellular carcinoma cells at clinical radiation doses ranging from 2 to 6 Gy (69). METTL3 inhibitors represented by STC-15 break the METTL3-inflammatory factor-METTL3 positive feedback circuit and restrain inflammatory activation when radiation doses fall between 2 and 10 Gy. Meanwhile, these compounds trigger tumor cell ferroptosis and strengthen the radiosensitivity of hepatocellular carcinoma (68). Therapeutic regimens integrating these two types of reagents produce dual beneficial outcomes, which include shielding normal hepatic parenchymal tissue and amplifying anti-tumor radiosensitization. Taken together, therapeutic strategies targeting METTL3 heavily rely on precise cell-type selectivity, while interventions against ALKBH5 place greater emphasis on rational temporal staging during disease progression.

## 5 Discussion and conclusion

Radiation-induced liver injury is a severe adverse complication arising from radiotherapy, and its pathological progression cannot be simply ascribed to DNA damage within hepatocytes. This disease arises from the pathological interplay between aberrant intercellular crosstalk and dysregulated m^6^A epitranscriptomic modification. These two tiers of regulatory machinery jointly drive the transition from acute intrahepatic inflammation to persistent hepatic fibrosis, and collectively determine the full pathogenic process and clinical prognosis of RILI (11). At the early phase of RILI development, ionizing radiation stimulates injured hepatocytes to release DAMPs. These molecules subsequently activate KCs and initiate a robust inflammatory cascade marked by massive pro-inflammatory cytokine secretion (24, 25). Multiple intricate positive feedback circuits have continuously amplified such inflammatory reactions. Combined with reciprocal signaling between M2 macrophages and HSCs, the overall hepatic microenvironment has undergone a fundamental phenotypic shift from acute inflammatory damage toward progressive fibrotic remodeling (31, 32).

METTL3 exhibits cell-type-specific expression changes after radiation exposure, with suppressed expression in irradiated hepatocytes (49) and elevated expression in KCs (54). These divergent regulatory profiles do not conflict with one another but instead constitute a specialized intercellular homeostatic circuit that shapes hepatic responses to radiation. Specifically, radiation-elicited hepatocyte injury stimulates the release of damage-associated molecular patterns such as HMGB1. These molecules activate KCs and elevate METTL3 abundance to facilitate TNF-α secretion (49, 54). Increased TNF-α in return modulates hepatocytic METTL3 levels to restrict unrestrained hepatocellular apoptosis (80). This regulatory circuit supports self-limited hepatic repair under balanced physiological conditions, yet sustained or hyperactivated METTL3 signaling within KCs has driven progressive pathological deterioration of RILI (34).

Cumulative preclinical evidence demonstrates that differential METTL3 expression exhibits a dose-dependent pattern under clinically relevant radiation doses (2–20 Gy). By contrast, ultra-low doses below 0.1 Gy cannot trigger this feedback cascade (76). Systemic modulation of METTL3 delivers unsatisfactory therapeutic outcomes because this broad intervention simultaneously disrupts two protective biological programs. One protective effect originates from physiological METTL3 suppression in hepatocytes that mediates anti-apoptotic activity, and the other stems from moderate innate immune activation induced by METTL3 upregulation in KCs (48, 51). For this reason, cell-type-selective targeting tactics have become essential for precise METTL3 intervention. Treatment regimens should preserve or amplify physiological METTL3 downregulation in hepatocytes to prevent overintervention during hyperinflammatory phases, while selectively blocking METTL3 enzymatic activity in KCs. Taken together, the divergent METTL3 expression signatures in hepatocytes and KCs have assembled a homeostatic negative feedback loop. Mechanistically, DAMPs secreted by irradiated hepatocytes activate KCs and upregulate METTL3 to initiate inflammatory cascades, and TNF-α produced by activated KCs in turn tunes hepatic METTL3 expression to curb excessive hepatocyte apoptosis.

During the acute inflammatory phase of RILI, radiation induces the upregulation of ALKBH5 in HSCs. ALKBH5 stabilized HMGB1 transcripts by mediating demethylation of the 3’UTR of HMGB1 mRNA, thereby activating the STING-IRF3 innate immune pathway and exacerbating radiation-induced sterile inflammation (52). In the chronic fibrotic phase, alongside reduced HMGB1 levels and progressive HSC activation, ALKBH5 facilitated NF-κB/Smad2 signaling activation and extracellular matrix deposition by demethylating TIRAP mRNA in HSCs (51). This functional substrate switching of ALKBH5 occurs in a time-dependent manner following irradiation, with distinct temporal windows corresponding to RILI progression. The HMGB1-targeted pro-inflammatory effect dominates within 1–3 days post-irradiation (acute phase), whereas the TIRAP-mediated pro-fibrotic function also dominates at 2–4 weeks after irradiation (chronic phase). Collectively, ALKBH5 exhibits prominent temporal functional specificity throughout RILI progression: in the acute stage it preferentially demethylates and stabilizes HMGB1 mRNA to initiate HSC-mediated inflammatory responses, while switching its target to TIRAP mRNA to drive fibrotic signaling in the chronic stage (51, 52).

These findings have suggested that temporal intervention precision outweighs cell-type selectivity for ALKBH5-targeted therapy. In the acute inflammatory stage, although ALKBH5 exacerbates hepatic inflammation, pharmacological inhibition of ALKBH5 may disrupt physiological damage signal transduction and inflammation resolution, thereby aggravating hepatocellular injury. In contrast, during the chronic fibrotic stage, ALKBH5 predominantly activates the NF-κB/Smad2 signaling axis via TIRAP mRNA demethylation in HSCs to promote hepatic fibrosis; thus, ALKBH5 inhibition at this stage can effectively halt fibrotic progression of RILI.

The dual regulatory roles of METTL3 across diverse liver injury models highlight its cell-type- and pathology-dependent functional specificity. The pro-inflammatory property of METTL3 in alcoholic hepatitis is primarily attributed to its modulation of pyroptotic signaling in Kupffer cells (81). In contrast, the hepatoprotective function of METTL3 during hepatic ischemia-reperfusion injury depends on HO-1-mediated cytoprotective cascades within hepatocytes (82). Such opposing phenotypic outcomes essentially originate from disparities in dominant effector cell populations and activated downstream signaling pathways under distinct pathological contexts. Furthermore, the profoundly different hepatic phenotypes observed in perinatal and adult Mettl3-knockout mice have validated the indispensable role of METTL3 in hepatic development and maturation (83). Nevertheless, liver homeostasis in mature hepatic tissues can be maintained independent of METTL3, which has indicated the existence of developmentally dependent epigenetic compensation mechanisms in the adult liver.

The functional heterogeneity of ALKBH5 across various hepatic fibrosis models reconciles the inconsistent conclusions reported in previous studies. In CCl_4_-induced liver fibrosis, ALKBH5 alleviates fibrotic progression by modulating mitochondrial dynamics through Drp1 m^6^A demethylation and inhibiting hepatic stellate cell activation via the YTHDF1-PTCH1 signaling axis (84). By comparison, ALKBH5 exhibits distinct cell-type-specific expression and functional patterns in nonalcoholic steatohepatitis (NASH)-associated fibrosis. Reduced hepatocytic ALKBH5 expression correlates with the progression of NAFLD-related fibrotic lesions, further illustrating the cell-specific functional traits of ALKBH5 in metabolic liver disorders (85). These findings have suggested that fibrotic stimuli derived from chemical toxicity or metabolic dysfunction fundamentally reshape the regulatory profile and substrate preference of ALKBH5, thereby demonstrating the intrinsic heterogeneity of m^6^A epitranscriptomic regulation in different fibrotic liver diseases.

Collectively, these advances have deepened the mechanistic understanding of epitranscriptomic regulatory mechanisms underlying liver injury and fibrogenesis. Rather than relying on universal therapeutic regimens, precise intervention strategies tailored to specific disease categories, pathological contexts and developmental stages are essential to achieve optimal clinical efficacy for liver disease treatment.

m^6^A modification displays distinct dose-dependent signatures in response to ionizing radiation. Low-dose radiation below 0.1 Gy triggers adaptive epitranscriptomic responses, while high-dose radiation exceeding 1 Gy induces persistent pathological alterations in global m^6^A modification landscapes (49). Nevertheless, such dose-effect relationships have remained insufficiently clarified within the RILI pathological context.

Although therapeutic strategies targeting m^6^A modification and intercellular crosstalk yield promising outcomes in preclinical animal models (62), multiple bottlenecks have still impeded their clinical translation. First, the safety profiles of combinatorial targeted therapies require comprehensive clarification. RILI pathogenesis relies on sophisticated pathological cascades that integrate intrahepatic inflammation, fibrotic remodeling and immune dysregulation, such that single-target interventions fail to fully reverse disease progression. Combinatorial blockade of chemokine signaling axes and core m^6^A regulators is hypothesized to disrupt pathological positive feedback loops through synergistic actions, whereas its clinical practicability relies on both stable therapeutic efficacy and controllable systemic safety. For example, the combination of novel m^6^A-targeted inhibitors and traditional radioprotectants, as well as nanomedicine-based regimens that simultaneously suppress pro-inflammatory cytokine signaling and specific m^6^A modification, may cause unforeseen drug interactions and adverse hepatic reactions (73). Second, *in vivo* tracking of exosome-based therapy still faces substantial technical obstacles. Current investigations focusing on exosome-mediated intercellular communication predominantly rely on *in vitro* co-culture systems (86), while only limited progress has been achieved via near-infrared-II fluorescence imaging approaches. Existing imaging techniques cannot precisely differentiate exosome internalization among hepatocytes, Kupffer cells and liver sinusoidal endothelial cells. This limitation hinders accurate evaluation of their *in vivo* distribution, uptake efficiency, and metabolic half-life (78). Third, optimal intervention time windows for RILI treatment have remained poorly defined. RILI progresses sequentially through acute inflammation and chronic fibrosis. During these stages, m^6^A modifying enzymes exert stage-specific and even opposing biological functions. Targeted inhibition of METTL3 ameliorates hepatic damage in the early inflammatory phase, whereas intact METTL3 activity is indispensable for hepatic regeneration during the subsequent tissue repair stage (49, 56). Furthermore, the rapid and dynamic progression of RILI blurs the boundary between inflammatory initiation and endogenous tissue repair. Systematic studies that calibrate precise administration timing for m^6^A-based targeted therapy have still lacked.

Future research priorities can be summarized based on the above translational limitations. Advanced liver-specific targeted delivery systems should be urgently developed. Systemic administration of current m^6^A modulators including STM2457 and CS2 carries potential systemic toxic risks. Engineered exosomes equipped with surface modification strategies such as CD44 targeting achieve precise liver and cell-specific drug delivery, thereby enhancing intrahepatic drug accumulation and alleviating off-target systemic adverse effects (62). However, their *in vivo* stability and delivery efficiency in human tissues have remained to be validated. Follow-up studies should systematically evaluate their pharmacokinetic characteristics and biosafety using large animal models instead of relying merely on *in vitro* cell experiments and murine data.

In addition, technical barriers restricting *in vivo* exosome tracing require breakthrough optimization. Future research can establish mouse models with fluorescently labeled exosome overexpression combined with recipient cell-specific reporter systems, such as HSC-specific fluorescent labeling, to directly trace the migratory trajectories and biological behaviors of therapeutic exosomes within intact hepatic tissues.

In summary, epitranscriptomic m^6^A regulation and intercellular crosstalk provide innovative mechanistic insights and potential therapeutic targets for RILI intervention. However, most evidence supporting this combined strategy remains at an early preclinical stage. Definitive conclusions regarding its efficacy in overcoming RILI are therefore not yet available. Several unresolved translational challenges remain to be addressed, including unclear safety profiles of combination regimens, technical bottlenecks in *in vivo* exosome tracking, and undefined stage-specific therapeutic windows. Further in-depth exploration of these issues is essential to determine whether such strategies can ultimately improve the long-term prognosis of patients with radiotherapy-associated hepatic injury.
